# Clinical Applications of Virtual and Augmented Reality in Radiology: A Scoping Review

**DOI:** 10.3390/jcm14207438

**Published:** 2025-10-21

**Authors:** Somin Mindy Lee, Henrique Coimbra Baffi, Tolulope Ola, Brian Tsang, Aaryan Gupta, Ricardo Faingold, Jennifer Stimec, Andrea S. Doria

**Affiliations:** 1 Department of Diagnostic and Interventional Radiology, The Hospital for Sick Children, Toronto, ON M5G 1X8, Canadatolulopeola@rcsi.ie (T.O.); ricardo.faingold@sickkids.ca (R.F.); jennifer.stimec@sickkids.ca (J.S.); 2 Department of Radiology, Ottawa University, Ottawa, ON K1N 6N5, Canada; brian.tsang@mail.utoronto.ca; 3 Department of Medical Imaging, University of Toronto, Toronto, ON M5T 1W7, Canada; 4 Research Institute, The Hospital for Sick Children, Toronto, ON M5G 1X8, Canada

**Keywords:** virtual reality, augmented reality, scoping review, preferred reporting items for systematic reviews and meta-analyses (PRISMA), medical field, clinical applications

## Abstract

**Background**: Virtual reality (VR) and augmented reality (AR) have emerged as innovative tools in healthcare, particularly using diagnostic and interventional imaging methods, offering new avenues for enhancing patient care and procedural outcomes. Their applications range from improving preoperative planning and pain management to providing advanced procedural support and training. Despite their growing integration into clinical practice, evidence of their cost-effectiveness and specific clinical benefits when using radiological tools remains limited. This review aims to map the current landscape of VR and AR applications using radiological modalities and highlight areas for future research. **Objective**: This scoping review explores the clinical applications of VR and AR in different radiological fields, aiming at assessing target areas, cost-effectiveness, and benefits of these technologies. **Methods**: We conducted a comprehensive literature search using the Preferred Reporting Items for Systematic Reviews and Meta-Analyses (PRISMA) framework. A total of 15 primary studies were included, covering diverse populations and applications of VR and AR. **Results**: In total, 15 studies (N = 781 patients) were included, with sample sizes ranging from 6 to 120. These studies highlighted various clinical applications of VR and AR, including imaging-guided preoperative planning, pain management, and procedural support. Although several studies demonstrated improvements in patient experiences and diagnostic accuracy, cost-effectiveness data were lacking. Notably, 47% of the studies focused exclusively on pediatric populations (N = 363), and 33% were randomized controlled trials. Quality assessment using the STARD criteria revealed that 60% of studies were rated as good (score > 12), 27% as fair (score 10–12), and 13% as suboptimal (score < 10), with inter-reader reliability showing substantial agreement (ICC = 0.76; 95% CI: 0.64–0.91). Out of 15 included studies, only 6 (40%) reported statistically significant improvements in patient experiences, with the remaining studies reporting positive trends (e.g., feasibility, usability, improved planning). Individual studies demonstrated significant benefits of VR interventions; for instance, one study reported a reduction in distress scores by a mean of 3.0 (95% CI: 1.0–5.0) and a decreased need for parental presence (risk ratio 0.3; 95% CI: 0.1–0.7; *p* < 0.001) compared to conventional methods. **Conclusions**: VR and AR technologies hold promise in enhancing patient care and procedural outcomes. Future research should focus on the cost-effectiveness of these technologies and identify specific target populations that would benefit the most. Additionally, adherence to the Standards for Reporting of Diagnostic Accuracy (STARD) guidelines should be encouraged to ensure transparent and comprehensive reporting in VR and AR studies.

## 1. Introduction

Virtual reality (VR) and augmented reality (AR) are technological advancements extensively used in entertainment, sports, gaming, and simulation [1]. VR and AR hold the potential to revolutionize how clinicians manage healthcare conditions and how patients undergo diagnostic testing and therapeutic procedures. These technologies can assist healthcare professionals in addressing cognitive challenges related to patient treatment and planning, managing phobias, alleviating pain, addressing other disease-related symptoms, and planning complex virtual surgeries [2], thereby enabling more cost-effective decision-making in patient care.

VR is characterized by immersing the user in a simulation, specifically in a three-dimensional computer-generated environment [3]. Within this environment, users have no visualization of the world outside the virtual environment; sensory input is provided through a head-mounted display, replacing the user’s native surroundings. As a result, users perceive themselves as experiencing a non-physical virtual world [3,4,5]. In contrast, AR does not entirely eliminate the real world from the user’s view [4]. Instead, virtual objects and structures are superimposed onto the “real world” via a head-mounted display or another type of interface, enabling users to interact simultaneously with both real and virtual elements [6,7].

Competitive markets and advances in hardware and software have driven down the costs of VR and AR technologies, facilitating their integration into various medical applications such as medical education, procedural planning, and therapeutic interventions.

Current and future applications of VR and AR include providing alternative approaches to 3D printing allowing radiologists to interact with images in a 3D format, aiding in a better informed decision-making process; (2) facilitating the understanding of anatomic relationships for diagnosis, interventional radiology procedures and surgical planning of complex health conditions; (3) assisting in real-time reconstructions (e.g., AR superimpositions onto patients during vascular interventions); (4) enhancing image interpretation in radiology reading rooms [8]; (5) serving as relaxation or distraction therapy to reduce pain and anxiety in patients, particularly as an alternative to medication [3]; (6) offering interactive VR and AR simulations for education and training on challenging anatomical concepts [4]; (7) expediting review and analysis of imaging data for clinical research [9]; (8) enabling remote monitoring and treatment of patients [9].

Despite the availability of scoping and systematic reviews on medical education applications of VR and AR [10,11,12] and the effect of VR on pain, anxiety, and fear among emergency department patients [13], and of papers on clinical applications of VR and AR in radiology on an unstructured or narrative basis [3,4,9,14], to our knowledge, no previous scoping or systematic review has specifically addressed their clinical applications in the pediatric or adult population. Of note, the pediatric population can particularly benefit from these technologies due to behavioral challenges during imaging.

The purpose of this scoping review is to assess the current state of knowledge on non-education-based applications of VR and AR in radiology in pediatric and adult populations. A comprehensive understanding of the current applicability of VR and AR in the medical field should guide further investigations into their value in specific areas of medicine, support the development of evidence-based guidelines, and highlight gaps in the literature concerning their use for both patients and healthcare professionals. Hence, the overarching study questions of this scoping review are:

1. What are the target areas of non-educational clinical applications of VR and AR in the pediatric and adult populations?
2. Are VR and AR cost-effective strategies in the clinical or surgical management of patients compared with traditional strategies?
3. What are the benefits of utilizing these novel technologies in the proposed population, including their impact on the accuracy of diagnostic tests?

## 2. Methods

The Research Ethics Board of our institution waived approval due to the public domain nature of the primary studies included in this review.

### 2.1. Study Design Framework

The Patient, Intervention, Comparison, and Outcome (PICO) framework and the Preferred Reporting Items for Systematic Reviews and Meta-Analyses (PRISMA-ScR) [15] were used to define the study cohort characteristics, study design, and methods. PRISMA-ScR is not a quality assessment tool, but instead it is designed to ensure transparent reporting in systematic reviews. The PICO eligibility criteria are summarized in Table 1. The Standards for Reporting of Diagnostic Accuracy (S) [16] were applied to assess the reporting quality of the literature included in this review. Although STARD is primarily for diagnostic accuracy, we selected it to assess reporting transparency across heterogeneous study designs. Items that were not applicable (e.g., reference-standard criteria) were marked N/A in the checklist.

### 2.2. Search and Data Collection Strategies

The initial electronic literature search was conducted by two independent reviewers (T.O. and H.B.) with the assistance of an experienced staff librarian (Q.M.) and was later updated by an independent reviewer (S.M.L.) with another staff librarian (J.C.). Studies were identified using a predefined list of search terms tailored for MEDLINE OvidSP (US National Library of Medicine, Bethesda, MD, USA), EMBASE (Elsevier, Amsterdam, The Netherlands), and the Cochrane Library from 1 January 2000 to 30 January 2025. Manual screening of reference lists from the included studies was also conducted.

**Figure 1:**
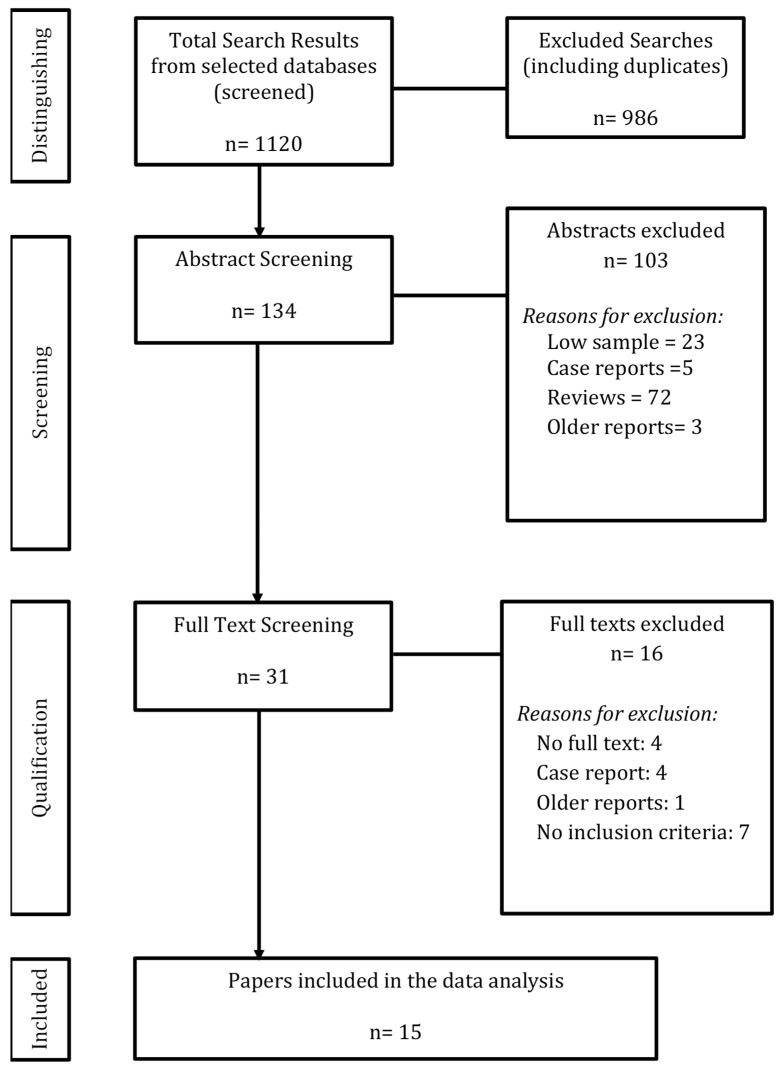
Literature search output and study identification.

Search terms were adapted for each database’s vocabulary, using combinations such as (virtual reality OR augmented reality) AND (diagnostic imaging OR radiology) AND (pediatrics OR infant OR child OR adolescent). Additional age-specific text search terms were applied. Details of the search strategy are available in Figure 1.

### 2.3. Study Selection Process

Titles and abstracts were independently screened for inclusion by three reviewers (T.O., H.B., and S.M.L.) to determine eligibility. Full articles were read by reviewers for final inclusion. Disagreements were resolved by a senior reviewer (A.S.D.), an experienced epidemiologist and radiologist with over 20 years of expertise, who acted as a tiebreaker.

Papers with consensus among reviewers were included in the review. Following the resolution of discrepancies, full-text articles of the selected abstracts were retrieved. These articles were then assessed using predetermined eligibility criteria. The initial batch was reviewed by T.O. and H.B., while the updated batch was reviewed by S.M.L., with calibration sessions involving A.S.D. for systematic consistency. Details of the selection process are illustrated in Figure 1.

### 2.4. Eligibility Criteria for Primary Studies

Studies were included if they met the following criteria: (a) clinical studies or case series with 6 or more subjects: (b) use of virtual reality as a clinical (e.g., pre-operative planning), interventional (e.g., as an analgesic or relaxation method, ultrasound-guided anesthesia), alternative therapy (e.g., as an alternative to 3D printing in radiology) or training tool to an imaging diagnostic test or imaging-guided procedure or that used imaging as an outcome measure; (c) be written in English, Spanish, Portuguese, French, German, Korean, or Italian; (d) be PubMed-indexed.

Exclusion criteria included non-human studies, publications prior to 2000 (to focus on newer technologies), abstracts from scientific meetings, editorials, personal opinions, letters to the editor, protocols, case reports, and reviews. We did not restrict inclusion by age group; both pediatric and adult clinical imaging applications of VR/AR were eligible. The fact that 7 of the 15 included studies (47%) focused exclusively on pediatric populations emerged post hoc during data extraction rather than being an a priori focus.

### 2.5. Data Extraction, Analysis and Critical Appraisal

Following the final selection, data extraction and critical appraisal were performed. The Preferred Reporting Items for Systematic Reviews and Meta-analysis (PRISMA) checklist [9] was used to guide the database of study characteristics for the included studies.

The Standards for Reporting of Diagnostic Accuracy (STARD) checklist [16] was used to assess reporting quality and methodology of selected primary papers. The original 2015 STARD [16] has a 34-point maximum for the sum of the 30 essential items (all articles receiving a score of 1), taking into account that items 10, 12, 13 and 21 are dichotomous (i.e., a, b for each item) and items 10b, 11, 12a/b, 13a/b; 14–17; 22; 23, 24, 25 are related to a reference standard which was not used in most primary studies of this review; a modified maximum score of 20 was used to assess reporting quality of included primary articles. Scores were categorized as follows: (a) good quality: >12; (b) fair quality: 10–12; (c) suboptimal quality: <10. The papers were independently assessed, reviewed and scored by the reviewers (S.M.L., T.O. and H.B). Any discrepancies in scoring, where consensus was not achieved, was discussed and resolved by the tiebreaker (A.S.D.).

### 2.6. Statistical Analysis

STARD scoring was designated as follows: 1, fully meets condition; 0.5, partially meets condition; 0, does not meet condition; N/A, not applicable. STARD scores were categorized as follows: (a) excellent quality: >15; (b) good quality: >12; (c) fair quality: 10–12; (d) suboptimal quality: <10.

STARD agreement levels were defined as: total agreement, identical scores by both reviewers; partial agreement, scores differed by 0.5 points (e.g., 0 and 0.5); no agreement, full score difference (e.g., 0 and 1). Inter-rater reliability was assessed using intraclass correlation coefficients (ICCs) to evaluate agreement between reviewers’ STARD scores. As per Altman et al. [18] based on 95% confidence intervals of ICCs, values >0.80 indicated excellent inter-reader reliability; values 0.61–0.80, substantial reliability; values 0.41–0.60, moderate reliability and values ≤0.40, poor reliability.

## 3. Results

### 3.1. Study Selection

The search yielded 1120 references, from which 986 duplicates and disparate studies were removed. A total of 134 studies underwent title and abstract screening. Of these, 103 were excluded for not meeting the inclusion criteria. Subsequently, 31 studies were assessed for full-text eligibility, and 16 were excluded as described in Figure 1. Ultimately, 15 studies were included in the scoping review: 9 (60%) focused on pre-operative planning and 6 (40%) on interventions. The 15 included primary studies encompassed a total of 781 patients. However, due to the lack of standardized reporting of demographic details, a comprehensive age analysis was not feasible. Only 60% of studies specified participant sex, and 0% reported ethnicity. The average, median, and range for age of patients of included primary studies are reported in this review as they appeared in the original articles.

The summary characteristics of the primary studies and participants are presented in Table 2, Table 3, Table 4 and Table 5.

### 3.2. Primary Studies’ Research Design

Most primary studies (33%) were randomized controlled trials (RCTs). Sample sizes across all studies were small (ranging from 6 to 120 patients). Furthermore, psychometric properties [18] were not applied to measurements or scales used in these studies.

Table 2, Table 3, Table 4 and Table 5 summarize the research designs and patient characteristics:

- Randomized controlled trials (RCTs), prospective design: 5 studies (33.3%) [22,24,25,27,33], including one single-blinded study [33].
- Descriptive/case series (6–10 patients): 5 studies (33.3%) [19,20,23,26,31].
- Protocol development/virtual modeling for specific clinical indications (>10 patients): 3 studies (20%) [29,30,32].
- Non-RCT cross-sectional study with a control group: 1 study (6.7%) [29].

There was substantial variability in study design, power calculations, and sample sizes across studies. The greatest variability was observed within case series, with sample sizes ranging from 6 patients in studies by Ieiri and Souzaki [19,20] to 120 participants in Ryu’s paper [25].

Seven of the 15 studies (47%) focused exclusively on pediatric populations (N = 363 patients), including studies by Ieiri et al., Souzaki et al., Zhao et al., Han et al., Milano et al., Stunden et al., Ryu et al. [19,20,21,22,23,24,25]. Patient ages ranged from 0.1 month to 15 years. However, demographic reporting was inconsistent, with some studies failing to mention patient sex [21,23,26,27,28].

### 3.3. Reporting Quality of Primary Studies (Using STARD)

The overall quality of reporting, as assessed using the STARD guidelines [16], was deemed good/acceptable. Of the studies included in this review, 27% were considered as presenting with excellent quality scores (scores > 15) [22,24,27,33], 40% as good/acceptable quality (scores > 12) [21,23,25,28,29,32], 20% were considered as of fair quality (scores ≥ 10 and ≤12) [19,26,30] and 13% were classified as presenting with suboptimal quality (scores < 10) [20,31] (Table 6).

Several sections of the primary studies were suboptimally reported, including intended sample size, flow diagrams, registration information, and full protocol access. Suboptimally reported sections included:

- Title or Abstract and Introduction: item 1: identification of the study as a diagnostic accuracy study using at least one measure of accuracy: no papers met this criterion.
- Methods (Study Design, Participants, Test Methods, Analysis): item 6: eligibility criteria: 4/15 studies scored 0; item 8: reporting where and when eligible participants were identified: 3/15 scored 0, and 2/15 scored 0.5; item 9: participant recruitment methods (e.g., consecutive, random, convenience): only 8/15 (53.3%) studies provided details; item 18: intended sample size and determination method: only 5/15 (33.3%) studies provided this information.
- Results (Participants, Tests): item 19: flow diagrams of participant selection: only 6/15 studies (40%) included diagrams; item 20: 10/15 (66.6%) scored 1, 2/15 (13.3%) scored 0.5 for demographic and clinical characteristics.
- Discussion: item 26: 2/15 (13.3%) papers scored zero on consensus for not adequately presenting study limitations.
- Other Information: item 28: 7/15 (46.7%) did not present a registration number or the name of a registry. This may be because only 5 papers (33%) were classified as clinical studies; item 29: only 4/15 (26.7%) studies mentioned where the full protocol could be accessed; item 30: 5/15 papers (33.3%) did not list sources of funding or other forms of support.
- The final STARD scores of the papers are presented in Table 6. Supplementary Table S1 shows the full STARD breakdown of items per study.

### 3.4. Inter-Reader Reliability of STARD Scoring

Inter-reader agreement for STARD scoring was substantial, with an ICC of 0.76 (95% CI, 0.64–0.91) for the 15 reviewed studies. The most frequent disagreements occurred on methods items—particularly STARD item 6 (eligibility criteria), item 8 (where and when participants were identified), and item 9 (recruitment methods)—each showing initial reviewer differences of 0.5 point. All were reconciled through consensus with the senior reviewer.

### 3.5. Summary of Data from Primary Studies

Out of 15 included studies, 6 (40%) reported statistically significant improvements in patient experiences (Han et al., 2019, [22]; Ryu et al., 2021, [25]; Yang et al., 2019, [33]; Nambi et al., 2020, [27]; Sadri et al., 2023, [28]; Chuan et al., 2023, [29]). The remaining 9 (60%) studies reported positive trends (e.g., feasibility, usability, improved planning) but without statistical testing or without reaching significance (Kockro et al., 2000, [30]; Hohlweg-Majert et al., 2005, [31]; Qui et al., 2010, [32]; Ieiri et al., 2012, [19]; Souzaki et al., 2013, [30]; Zhao et al., 2015, [21]; Simpfendorfer et al., 2016, [26]; Milano et al., 2021, [23]; Stunden et al., 2021, [24]). Specifically, this scoping review aimed to address the following overarching questions:

***Target areas of clinical applications of VR and AR in the pediatric and adult populations:***

Clinical applications of VR and AR reported in the primary studies primarily related to pre-surgical planning.

In pediatric populations, Milano et al. [23] explored the advantages of enhanced 3D visualization for complex cardiac surgery planning, Han et al. [22] and Ryu et al. [25] each showed that immersive VR education can reduce pediatric anxiety and procedural times during chest radiography.

In adult populations, Kockro et al. [30] tested new intraoperative software. In more recent investigations, Chuan et al. [29] designed a high-fidelity VR trainer for teaching ultrasound-guided regional anesthesia skills to adults.

Evaluating both children and adults, Qui et al. [32] assessed the effectiveness of image processing in pre-surgical planning. Whereas Zhao et al. [21] reported on the intraoperative support provided by VR in patients aged 1–20 months, Simpfendorfer et al. [26] did so in adults. As direct clinical interventions, VR was compared to conventional therapies in studies by Yang et al. and Nambi et al. [27,33]. Yang evaluated adolescents and adults (age range, 14–62 years) in the VR group and young to senior adults (age range, 20–65 years) in the non-VR group. Nambi, on the other hand, assessed young adults (age range, 18–25 years) only, whereas Sadri et al. [28] further demonstrated the potential of AR overlays to reduce contrast use during cerebral embolic protection in TAVR without prolonging the procedure.

Concerning the overarching question #1 of this scoping review (“What are the target areas of non-educational clinical applications of VR and AR in the pediatric and adult populations?”), the studies of this review employed different methods for planning, patient preparation and procedural guidance.

**1. Planning And Pre-Operative Visualization**

Primary studies of this review demonstrated how VR/AR enhanced pre-surgical planning by improving visualization of complex anatomy. In children and adults, Kockro et al. [30] applied the costly VIVIAN VR system for neurosurgical planning, enabling intuitive spatial navigation but requiring significant resources. Similarly, in a mixed aged population (patients aged 4–70 years), Qui et al. [32] used VR stereoscopic tractography for glioma surgery, achieving trajectory optimization and safer resections and Hohlweg-Majert [31] applied VR in craniofacial reconstruction across a broad mixed population (infants to adults). In pediatric populations, Milano et al. [23] showed that VR provided more cost-effective visualization compared to 3D printing for congenital cardiac surgery.

**2. Patient Preparation And Anxiety Reduction**

The results of this review showed that pediatric patients benefited most clearly from VR interventions aimed at anxiety reduction, while adult populations showed improvements in surgical anxiety and rehabilitation support. Immersive VR interventions consistently reduced anxiety and improved patient experiences, particularly in pediatric radiology. Han et al. [22] and Ryu et al. [25] demonstrated statistically significant reductions in distress during pediatric chest radiography, with shorter procedure times and less parental presence required. Yang et al. [33] reported similar benefits in a mixed aged population of children and adults undergoing arthroscopic knee surgery, with improved preoperative anxiety scores. Nambi et al. [27], on the other hand, used VR rehabilitation in young adults with chronic low back pain, reporting both imaging and biochemical improvements.

**3. Procedural Support And Training**

VR and AR provided tangible procedural benefits across populations. Pediatric applications focused on intraoperative visualization, while adult applications extended to interventional radiology and anesthesia training. In primary studies of this review, AR and VR systems were applied to real-time procedural support and skill training. Souzaki et al. [20] used AR overlays in pediatric oncology to assist with tumor localization during surgery. Stunden [24] and Chuan [29] developed VR training systems for ultrasound-guided regional anesthesia, showing performance improvements in both novice and experienced adult clinicians.

***Cost-effectiveness of VR and AR in clinical or surgical management of patients compared to conventional strategies***

While most studies of this scoping review did not provide formal economic analyses, four studies offered insights into cost implications of VR or AR in clinical applications.

Sadri et al. [28] examined the cost-effectiveness and procedural improvements offered by AR during cerebral embolic protection (CEP) in transcatheter aortic valve replacement (TAVR) of adults. The study demonstrated that AR significantly reduced the need for contrast angiography during procedures, resulting in lower contrast volume usage without increasing procedure times. The reduction in contrast used also minimized the risks of contrast-induced complications, such as nephropathy, providing additional long-term health benefits. The authors emphasized that while AR systems may involve substantial initial investment, the reduction in contrast usage, procedure times, and improved physician confidence make AR a cost-effective solution in procedural interventions.

Certain operating systems, such as the Virtual Intracranial Visualization and Navigation (VIVIAN) System used in Kockro et al.’s study of children and adults [30], provided advanced visualization but was resource-intensive and costly due to specialized equipment and programming. Conversely, Yang et al.’s study of children and adults [33] utilized commercially available head-mounted displays (HTC VIVE), which are head-mounted hardware and software displays/systems, more affordable financially. These systems have become increasingly less expensive since the time of the study’s publication, potentially enhancing their accessibility for broader clinical use. Milano et al. [23] commented that VR was less costly than 3D printing for complex congenital cardiac surgical planning in a pediatric population. Therefore, cost-effectiveness varied, with commercial VR showing overall a lower cost potential compared to specialized platforms.

***Benefits of using VR and AR, including implementation of the accuracy of diagnostic tests***

Nambi et al. stated that “virtual reality training is widely used because it reduces the difficulty of rehabilitation and increases participant safety” [27]. Yang et al.’s study reported significant improvements in APAIS (Amsterdam Preoperative Anxiety and Information Scale) scores for assessing preoperative anxiety in the VR group compared to the control group [33]. Furthermore, studies by Ieiri, Souzaki, and Milano [19,20,23] demonstrated the benefits of VR-assisted surgical planning. Their findings highlighted accurate identification, successful imaging guidance, and precise resection planning facilitated by VR.

The VR studies described in the primary papers of this review encompassed diverse applications, ranging from clinical interventions aimed at improving patient care to educational programs for training and skill development. Similarly, AR studies revealed versatile applications, with a strong focus on enhancing clinical procedures and providing educational guidance across various medical and surgical contexts. This was compared to conventional training exercises combined with Swiss ball rehabilitation over a four-week period. Zhao et al. [21] evaluated MR virtual endoscopy (MRVE), a virtual imaging technology that enables the visualization of the inner surface of lumina. MRVE was used to identify pathological changes in the cerebral ventricles and measure lumen diameters in hydrocephalus patients. Simpfendorfer et al. [26] employed intraoperative cone-beam CT images with AR guidance to ensure safe resection lines in laparoscopic partial nephrectomy procedures. Yang et al. [33] introduced a preoperative VR experience using 3D-reconstructed knee MRIs for patients undergoing arthroscopic knee surgery. This intervention reduced preoperative anxiety and improved patient satisfaction, underscoring the clinical utility of VR in enhancing the preoperative care experience.

Nambi et al. [27] implemented balance exercise training using the ProKin system, focusing on core stability in university football players with chronic low back pain. Beyond these examples, Chuan et al. [29] developed a high-fidelity VR trainer for ultrasound-guided regional anesthesia (UGRA), demonstrating improved skill acquisition among novice practitioners and reinforcing VR’s potential to enhance procedural accuracy and confidence. Han et al. [22] and Ryu et al. [25] similarly showed that immersive VR education decreases anxiety and distress in pediatric patients undergoing chest radiography, thereby reducing procedure time and potentially improving the quality of imaging. Meanwhile, Sadri et al. [28] highlighted how AR overlays can offer precise visualization of patient-specific anatomy in TAVR procedures, minimizing contrast usage without increasing procedure time. Collectively, these findings underscore the significant benefits of VR and AR in achieving more accurate diagnostics, improved safety, and higher procedural efficiency.

## 4. Discussion

### 4.1. Summary of Evidence

This scoping review identified published literature on the use of VR and AR as clinical or surgical imaging-based aid tools, for both pediatric and adults populations. Several studies highlighted the potential of these immersive technologies to enhance surgical planning, reduce patient anxiety, and improve procedural efficiency.

### 4.2. Population

Different types of treatments applied virtual reality experiences. Hohlweg-Majert et al. in Navigational Maxillofacial Surgery Employing Virtual Models [26] assessed the benefit of using VR in patients ranging from 3 weeks to 80 years. This article specifically informed readers on how virtual reality reacts in a preoperative environment and as an intraoperative navigational tool. Most of our research had no intention of restricting themselves to specific populations or age categories since we wanted to learn about the benefits of using this unique technology. Because we only found fifteen papers that addressed the research questions of this scoring system, we concluded that VR and AR are at critical stages of evaluation compared to existing technologies. There are many factors to consider when identifying target audiences, evaluating outcomes such as patient health and clinician benefits, and assessing the feasibility and resources for translation and implementation into clinical practice. As a result, we felt it was critical not to limit our target group, and we thought it was necessary to consider the total influence of VR and AR on all populations.

### 4.3. Improvement of Population Health and Experiences of Care

Our scoping study assessed new services that are now available due to advances in virtual reality technology, which may help all physicians to deal with complex surgeries. VR can facilitate the performance of procedures for distal radius fractures which are typically challenging. VR/AR allows the surgeon to do a more effective procedure because it affords a better view of the distal radius, which has a small diameter. The use of virtual reality increased the surgeon’s anatomic prediction capability as anticipating the final placement of the distal radius post osteotomy favored the use of a CT virtual reality environment [34]. The improved patient experience in alleviating pain and anxiety in procedures is demonstrated. It will be important in the future to create more objective criteria guidelines concerning patients’ experiences during VT/AR procedures for different age groups, including patients for whom VR can be challenging to tolerate such as older patients and persons with phobias. Additionally, VR has been shown to alleviate pain or anxiety, an effect that Han et al. [22] and Ryu et al. [25] both demonstrated in pediatric patients undergoing radiographic exams. By immersing children in a virtual environment before or during a procedure, these studies reported less distress, shorter procedure times, and higher patient (and parental) satisfaction.

From a training standpoint, Chuan et al. [29] showed that novice anesthesiologists learning ultrasound-guided regional anesthesia via VR-based training could achieve more accurate skill acquisition and improved procedural confidence. Collectively, these examples suggest that VR or AR can improve health outcomes and care experiences by fostering higher provider proficiency and supporting patients whether by lowering anxiety in children or boosting operator skill in complex tasks. However, future research should establish objective guidelines on how VR/AR influences patient and provider experiences, with attention to populations (e.g., older adults, patients with phobias) who might find immersive technologies more challenging.

Based on the target areas of clinical applications of VR/AR of this review, the information from primary studies focused more on the benefit of VR utilization from the physician’s perspective as they would be able to better understand the 3D reconstructed MR images.

Yang et al. [33] looked at the effect of VR experience in 3D reconstructed magnetic resonance images on anxious patients undergoing arthroscopic knee surgery. Because most adults realize the realities of undergoing surgery, this form of virtual reality usage would be more acceptable as a pediatric intervention. Nambi et al. [27] observed a study in which the age range was 18–25 years old and determined the radiological and biochemical effects of VR training in football players with persistent back pain. This is an example of proper VR use in a young-adult population, where VR is used to meet the needs of this specific population. Finally, Qui et al. [32] examine the potential clinical applications of presurgical 3D environments in patients with brain gliomas near motor circuits. The participants in this study ranged from 4 to 70 years old. The average age, however, was 46 (+/−17.98). This study is an excellent example of VR being used in people who have more severe acquired health consequences due to simply living longer. Therefore, our broad target group enabled us to examine the benefits of this unique technique across all specified demographics. However, future studies must define whether VR would be more effective for specific populations.

### 4.4. VR Technology

Few research papers suggested whether or not VR was a cost-effective intervention choice during our scoping analysis. For example, the VR technology utilized in Kockro et al. [30] was VIVIAN (Virtual Intracranial Visualization and Navigation). The VIVIAN system enables users to interact with complex imaging data rapidly, completely, and intuitively; nevertheless, when discussing the VIVIAN system’s cost, the study revealed that it was a costly operating system. Milano et al.’s study [23] also mentioned need for cost-effect evaluation in the future but deemed VR costs to be lower than 3D printing in pre-surgical planning. All the other studies made no mention of how VR affects expenses or how the VR system can help to lower intervention costs. As a result, given that the bulk of the studies we discovered focused on VR intervention, we propose that future research and development should concentrate on the cost of VR. For example, VR and AR anxiety reduction equipment, or cost-effective presurgical technology that can replace existing high-maintenance equipment, are intriguing research avenues to study.

### 4.5. Standards for Reporting Diagnostic Accuracy (STARD) of Primary Studies

Our review found modest compliance with the STARD checklist, with only 19/34 (56%) of checklist sub-items being reported across studies. Since most primary papers did not follow traditional randomized controlled trial designs or include a reference standard for estimation of diagnostic accuracy indexes, most studies lacked reference standards in their design, precluding the assessment of STARD sub-items related to a reference standard (test methods: 10b, 11, 12a/b, 13a/b; analysis: 14–17; results (participants): 21b; 22; results (test results): 23, 24, 25) which were deemed inapplicable to the analysis of the data of primary studies of this review.

Additionally, many studies failed to report variability between VR and non-VR groups (item 17) or adverse events (item 25). These gaps highlight challenges in identifying biases and ensuring transparency in research reporting. Improved adherence to STARD guidelines is necessary to reduce bias and enhance replicability and peer approval of future studies.

Some of the included studies lacked rigorous research designs, such as reference standards for assessment of diagnostic test accuracy, limiting the assessment of STARD items. Furthermore, the restricted sample sizes and case series’ characteristics of many of the included studies reduced the reliability and external validity of conclusions. The considerable variability in statistical methodologies also hindered result aggregation. Approximately 40% of studies exhibited fair or suboptimal reporting quality, highlighting the need for more robust research design in future studies. The shift from case series and personal experiences toward robust clinical trials with rigorous methodology is vital for advancing the field.

## 5. Limitations

This scoping review is subject to several limitations. Although we screened 1120 records, ultimately only 15 studies met our predefined eligibility criteria. This small sample size limits the breadth and generalizability of our findings and likely reflects both the novelty of clinical VR/AR applications in radiology and our strict inclusion criteria. We searched only three databases (MEDLINE OvidSP, EMBASE, and the Cochrane Library) and did not include gray literature or trial registries, so some relevant studies may have been missed. The fact that the primary studies had heterogeneous research designs ranging from randomized clinical trials to small case series made the synthesis of this scoping review challenging and a meta-analysis of data not feasible. Further, demographic reporting was incomplete, with only 60% of studies reporting participant sex, none reporting ethnicity, and an inconsistent presentation of age data, precluding meaningful subgroup analysis. As reported by the authors, 40% of studies lacked flow diagrams, 47% did not provide a trial registration number and one-third did not report funding sources. Inclusion/exclusion criteria and recruitment methods of selected primary papers were often unclear and psychometric properties of the scales used were not validated or reported. Our language restriction (English, Spanish, Portuguese, French, German, Korean, Italian) introduces potential publication bias. We limited our search to publications through 30 January 2025, so very recent work is not captured. Finally, we did not perform a formal risk-of-bias assessment beyond reporting transparency, which constrains our ability to evaluate methodological quality across heterogeneous study designs.

## 6. Future Directions

Although current studies demonstrate promise for VR/AR in procedural planning, anxiety reduction, and training, the field remains nascent. Going forward, larger multicenter randomized trials are needed to confirm clinical efficacy and to embed rigorous cost–utility analyses alongside patient-level outcomes. Standardizing demographic and reporting elements, using PRISMA-ScR for scoping reviews and STARD for diagnostic accuracy studies, will allow consistent capture of age, gender, ethnicity, and comorbidity data, and improve comparability across studies.

Research should also explore the usability and tolerability of immersive systems in older adults and patients with anxiety disorders to identify adoption barriers. Limited available data from this review suggest that VR/AR cost varies by platform. This highlights a critical research gap requiring robust economic evaluation.

Long-term follow-up is essential to assess skill retention, sustained anxiety reduction, effect on reduction in time of scanning for radiation-bearing imaging techniques and downstream overall healthcare utilization. Finally, implementation science frameworks should be applied to understand real-world barriers (IT infrastructure, staff training needed for staff adoption, workflow integration, reimbursement with the caveat of equipment cost variability, from commercial headset to specialized systems, patient tolerance issues in older adults and phobic individuals [14]), and emerging extensions such as mixed-reality, haptic feedback, and remote telementoring warrant evaluation to expand the clinical utility of VR/AR beyond current use cases.

## 7. Conclusions

Despite the methodological limitations of this scoping review, our findings suggest that VR holds significant potential to improve medical care. VR enhances imaging quality compared to traditional tools and offers benefits such as reduced preoperative anxiety and improved surgical outcomes. However, insufficient data exists to confirm VR’s cost-effectiveness, STARD guidelines should be more consistently followed to enhance the methodological quality of future studies, and the identification of target populations remains an area for further scientific exploration. Future clinical trials investigating new indications for VR/AR use to address gaps in the current literature and evaluating the cost-effectiveness of VR compared to other imaging and sedation techniques, particularly in vulnerable populations, such as children and older adults, are key for the advancement of the field.

## Figures and Tables

**Table 1:** PICOS eligibility criteria for included studies.

Element	Inclusion	Exclusion
Population	Human patients (all ages) undergoing any clinical or interventional radiology procedure with VR/AR	Non-human studies; healthy-volunteer studies without clinical imaging context
Intervention	Virtual reality (fully immersive via head-mounted display) or augmented reality (digital overlays) for clinical purposes (pre-op planning, procedural support, pain/anxiety management)	Educational-only VR/AR without clinical outcomes; 360° video without interactive tracking
Comparator	Standard care or preparatory methods (verbal instruction, tablet video, standard imaging)	No-comparator single-arm simulations
Outcomes	Clinical outcomes (anxiety/distress scores, procedure time, diagnostic accuracy), reporting quality (STARD score, ICC)	Pure usability metrics or developer-focused performance measures without clinical or reporting outcomes. Clinical outcomes (anxiety/distress scores, procedure time, diagnostic accuracy), reporting quality (STARD score, ICC)
Study Design	RCTs, cohort studies, case series with ≥6 participants	Case reports (<6 subjects), editorials, reviews, protocols, letters, conference abstracts
Language	English, Spanish, Portuguese, French, German, Korean, Italian	Other languages
Date range	January 2000–30 January 2025	Publications before January 2000 o4-mini-high

We defined virtual reality (VR) and augmented reality (AR) per Milgram and Kishino’s Reality–Virtuality continuum [17]: VR as fully immersive environments delivered via head-mounted displays, and AR as real-world overlays of digital content. Applications such as 360° video without interactive tracking were excluded/included as VR.

**Table 2:** Study and Patient Characteristics.

First Author	Year of Publication	Country of Origin	Number of Patients	Mean Age (SD) Years)	Median Age (Range) Years	% of Male (Rounded)	Prospective vs. Retrospective Design
Pediatric only (N = 7)							
Ieiri, S. [19]	2012	Japan	6	n/a	n/a (6–15)	n/a	Prospective
Souzaki, R. [20]	2013	Japan	6	n/a	n/a (1–12)	n/a	Prospective
Zhao, C. [21]	2015	China	30	n/a	n/(0.1–1.7) 1–20 Months	47%	Prospective
Han, S-H [22]	2019	Korea	99 in total Virtual reality group: 49; usual care group: 50	VR group: Mean 5.8 (SD, 1.3 years); Control group: Mean 5.6 SD, 1.2 year)	4–8 years	VR group: boys: 32 (65.3%); control group: boys: 26 (52.0%)	Prospective
Milano, E.G. [23]	2021	UK	10	n/a	n/a (0–1.1) 0.1–13 months	n/a	Retrospective
Stunden, C. [24]	2021	Canada	92	n/a	n/a (4–13)	61%	Prospective
Ryu, J-H [25]	2021	Korea	120 in total Virtual reality group: 60 tablet video group: 60		VR group: Mean 6.0 (4.8–7.0 years); tablet video group: Mean 6.9 (5.0–7.3)	VR group: boys: 25 (41.7%); tablet video group: boys: 32 (53.3%)	Prospective
Adult only (N = 4)							
Simpfendorfer, T. [26]	2016	Germany	10	n/a	n/a (38–69)	70%	Prospective
Nambi, G. [27]	2020	Saudi Arabia	36	n/a	n/a (18–25)	n/a	Prospective
Sadri, S. [28]	2023	United States	24	n/a	AR guidance group: 84 (79–87), control group: 75(73–85)	AR guidance group: 7 (58%), control group: 8 (66.7%)	Prospective
Chuan A [29]	2023	Australia, U.K.	21 novice and 15 experienced participants	Novice: Mean 21 (SD, 1) Experienced: Mean 41 (SD, 6)		Novice: Males 7 (33.3%); Experienced: 10 (66.7%)	Prospective
Pediatric and adult (N = 4)							
Kockro, R.A. [30]	2000	Singapore	21	n/a	n/a (11–66)	48%	Retrospective
Hohlweg-Majert, B. [31]	2005	Germany	107	n/a	n/a (0.1–80) 0.1 year = 3 weeks	n/a	Prospective
Qiu, T.M. [32]	2010	China	45 (baseline); 40 (follow-up)	46.0 (17.98)	n/a (4–70)	58%	Prospective
Yang, J.H. [33]	2019	South Korea	48	n/a	n/a Group VR: (15–62); Group Non-VR (20–65).	0 (all females)	Prospective

**Table 3:** Intervention details and Key findings.

First Author	Field	Diagnosis	Key Findings
Pediatric only (N = 7)			
Ieiri, S. [19]	Hematology	Hematological disorders: hereditary spherocytosis (HS) and idiopathic thrombocytopenia purpura (ITP)—treated by laparoscopic splenectomy with one patient also requiring a laparoscopic cholecystectomy	The proposed navigation system provides real-time anatomical information that cannot be obtained without the system. Registration accuracies for the study cases were acceptable in clinical operation settings.
Souzaki, R. [20]	Oncology	Pediatric malignancy (abdominothoracic)	The proposed AR navigation system was useful for detecting the tumor location during pediatric endoscopic surgery.
Zhao, C. [21]	Neurosurgery	Hydrocephalus/Intracranial pathology: clinical manifestation of enlarged head circumference, increased intracranial and growth retardation	“MR virtual endoscopy provides a non-invasive diagnostic modality that can be used as a supplemental approach to ventriculoscopy. Requires future studies to assess sensitivity and specificity of the technique.”
Han, S-H [22]	Radiology	Training of experimental group with a 3 min immersive virtual reality experience of the process of chest radiography guided by animation and narration.	During chest radiography, distress scores were lower in the treatment group, with a mean difference of 3.0 (95% CI 1.0 to 5.0). Compared with the control group, the virtual reality group had fewer children classified as distressed (score _5) (risk ratio 0.3, 95% CI 0.1 to 0.7), less need for parent presence (risk ratio 0.3, 95% CI 0.1 to 0.9), higher parental satisfaction scores (MD 20.8, 95% CI 21.5 to 20.1) and reduced procedure time (MD 19.9 s, 95% CI 6.6 to 33.3).
Milano, E.G. [23]	Cardiology	DORV and complex interventricular communications	3D pdf reached only 70%, whereas it was 95% after VR. Virtual reality can enhance understanding of suitability for biventricular repair in patients with complex DORV if compared to cross-sectional images and other 3D modeling techniques.
Stunden, C. [24]	Anesthesiology	examine the effectiveness of virtual reality (VR) simulation training in ultrasound-guided regional anesthesia.	VR-based simulation significantly improves clinical performance in ultrasound-guided regional anesthesia compared to standard training methods.
Ryu, J-H [25]	Radiology	Evaluated the effect of VR, compared with standard video, on reducing anxiety and distress in pediatric patients undergoing chest radiography.	The number of less distressed children (OSBD score < 5) was significantly higher in the VR group than in the tablet group (49 [81.7%]) vs. 32 [53.3%]) (*p* = 0.001). The OSBD scores, the need for parental presence, the procedure time, and the number of repeated procedures were all lower in the VR group.
Adult only (N = 4)			
Simpfendorfer, T. [26]	Urology	Renal malignancy (with complex or endophytic tumor localization)	Use of CBCT is feasible and safe and allows for direct access to complex or endophytic renal masses.
Nambi, G. [27]	Physiatry	Chronic low back pain	Positive effects of VR training on imaging and biochemical aspects of study subjects.
Sadri, S. [28]	Anesthesiology	Evaluating the effectiveness of training methods. Specifically, the training of clinicians in performing ultrasound-guided regional anesthesia, assessing whether virtual reality (VR)-based simulation can improve their skills compared to traditional training methods.	VR-based simulation training is as effective as standard training methods, showing no significant difference in the clinical performance outcomes between the two groups.
Chuan A [29]	Radiology	Training of an interventional procedure using ultrasound-guidance	The virtual reality trainer was comparable in terms of performance to other high-fidelity virtual reality software, possibility to act and quality of interface subscales (all *p* > 0.06), but not in the possibility to examine and self-performance subscales (all *p* < 0.009). It created workloads similar to those reported in real-life procedural medicine (*p* = 0.53).
Pediatric and adult (N = 4)			
Kockro, R.A. [30]	Neurosurgery	Intracranial pathology: complex neurosurgical anatomy of intra- and extra-axial brain tumors and vascular malformations.	The VIVIAN system allows users to work with complex imaging data in a fast, comprehensive, and intuitive manner but still requires a costly operating system. VIVIAN system substantially contributed to surgical planning by (1) providing a quick and better understanding of intracranial anatomic and abnormal spatial relationships, (2) simulating the craniotomy and the required cranial base bone work, and (3) simulating intraoperative views.
Hohlweg-Majert, B. [31]	Craniofacial	Craniomaxillofacial malformation	Author’s opinion-based without data analysis: Image-guided treatment improves preoperative planning by visualizing the individual anatomy and outlining the intended reconstructive outcome. Intraoperative navigation makes tumor and reconstructive surgery more reliable by showing safety margins, saving vital structures, and leading the reconstruction to preoperatively planned objectives.
Qiu, T.M. [32]	Neurosurgery	Cerebral Glioma	VR-based 3D stereoscopic tractography visualization enhances understanding of anatomic information of intra-axial tumor contours and adjacent PT, and results in surgical trajectory optimization initially and maximal safe tumor resection finally. Surgeons can predict the long-term motor functional outcome based on EPT increasing amplitude.
Yang, J.H. [33]	Orthopedics	Knee Pathology: Ligament rupture, Meniscus tear, Chondral lesion	Positive effects of VR experience of 3D reconstructed knee MRIs in patients undergoing arthroscopic knee surgery reducing anxiety around surgical encounters.

**Table 4:** Study Design and Outcomes.

First Author	Year of Publication	Number of Timepoints	Imaging Modality	Comparators	Key Findings
Pediatric only (N = 7)					
Ieiri S [19]	2012	Single	Multi-detector low CT (MDCT) (Aquilion 64, Toshiba Medical Co., Ltd., Tokyo, Japan)	n/a	The proposed navigation system provides real-time anatomical information that cannot be obtained without the system. Registration accuracies for the study cases were acceptable in clinical operation settings.
Souzaki R [20]	2013	Single	MDCT (multi-detector computed tomography) MRI	n/a	The proposed AR navigation system was useful for detecting the tumor location during pediatric endoscopic surgery.
Zhao C [21]	2015	Single	MRI (3D-T1 weighted, FSPGR; Signa EXCTTE 1.5 T MR machines (GE, USA), processing software (ADW 4.3) CT ventriculography [OPTIMN CT660 (128T) machine (GE, USA)]	Ventriculoscopy or CT imaging reference standard)	“MR virtual endoscopy provides a non-invasive diagnostic modality that can be used as a supplemental approach to ventriculoscopy. Requires future studies to assess sensitivity and specificity of the technique.”
Han, S-H. [22]	2019	Single (during chest radiography, after VR or verbal instruction intervention).	Chest radiography (x-ray)	Control Group: Received simple verbal instruction before chest radiography. VR Group: Received a 3 min immersive VR education explaining the radiography process.	VR education significantly reduced anxiety and distress in pediatric patients undergoing chest radiography. Lower distress scores in the VR group (OSBD score: 2.0 vs. 5.0 in control, *p* = 0.004). Lower need for parental presence (16.3% in VR groups vs. 36.0% in control) Shorter procedure time (55.1 s in VR group vs. 75.0 s in control) Higher parental satisfaction (9.4 in VR group vs. 8.6 in control) Fewer repeated procedures in the VR group (8.2% vs. 16.0%)
Milano, E.G. [23]	2021	Single	CMR, cardiac magnetic resonance; CT, computed tomography	n/a	3D pdf reached only 70%, whereas it was 95% after VR. Virtual reality can enhance understanding of suitability for biventricular repair in patients with complex DORV if compared to cross-sectional images and other 3D modelling techniques.
Stunden, C. [24]	2021	Dual	Ultrasound	virtual reality (VR)-based simulation training vs. standard training method	virtual reality (VR)-based simulation training was as effective as standard training methods for ultrasound-guided regional anesthesia. The study found no significant difference in the clinical performance between the two groups, suggesting that VR-based training can be a viable alternative to traditional training methods for developing skills in this area.
Ryu, J-H [25]	2021	Single (during chest radiography, after the VR or tablet video intervention)	Chest Radiography (X-ray)	Tablet group: watched a 3 min educational video on a tablet explaining chest radiography VR group: received the same educational content in an immersive VR environment using a head-mounted display	VR education significantly reduced distress and anxiety in pediatric patients compared to tablet video education Lower distress scores in the VR group (OSBD score: 1.0 vs. 4.0 in the tablet group, *p* < 0.001) Lower OSBD scores in the VR group (median 1.0) vs. the tablet group (median 4.0) (*p* < 0.001) Lower need for parental presence (8.3% in the VR group vs. 31.7% in the tablet group, *p* = 0.001) Shorter procedure time (48s in the VR group vs. 65s in the tablet group. *p* < 0.001) Easier procedure for radiology technologists in the VR group (ease-of-procedure rating 10.0 vs. 8.0, *p* < 0.001) Fewer repeated chest radiographs in the VR group (3.3%) vs. the tablet group (10%)
Adult only (N = 4)					
Simpfendorfer T [26]	2016	Single	CB-CT and fluoroscopy infusion	Nephrometry scores of tumors	Use of CBCT is feasible and safe and allows for direct access to complex or endophytic renal masses.
Nambi G [27]	2020	Dual (Baseline and after 4 weeks)	3-T MRI Ultrasound Device (Hitachi Ultrasound, Tokyo, Japan)	Immunosor bent assay (ELISA)	Positive effects of VR training on imaging and biochemical aspects of study subjects.
Sadri, S [28]	2023	Dual	CT and fluoroscopy	AR guidance vs. standard guidance	AR guidance significantly reduced contrast volume used during device deployment without increasing the time required for filter placement or fluoroscopy time. The AR system facilitated easier device placement and increased confidence in navigating the aortic arch during the procedure.
Chuan, A [29]	2023	Single (within this session, each participant performed 40 needling attempts across four different virtual nerve targets	Ultrasound	Novice group (participants with no prior experience in ultrasound-guided regional anesthesia (UGRA) vs. experienced group: participants with at least 50 ultrasound guided regional anesthesia procedures performed	Experienced participants performed slightly better than novices (*p* = 0.002) Performance scores remained above 80% for experienced participants from the third attempt onward, while novices showed more variability. VR trainer immersion was comparable to other high-fidelity VR software in realism, quality of interface, and possibility to act (*p* > 0.06). The VR trainer imposed workloads similar to real-life procedural medicine (*p* = 0.53) Novices exhibited greater initial learning improvements compared to experienced participants, who had more stable performance.
Pediatric and adult (N = 4)					
Kockro R, A. [30]	2000	Single	MRI (Siemens Expert 1.0-T unit, Stuttgart, Germany; General Electric Signa 1.5-T unit, Milwaukee, WI, USA), MRA, MRV venography, CT imaging (GE scanner; Picker 2000 scanner, Picker, Cleveland, OH, USA)	n/a	The VIVIAN system allows users to work with complex imaging data in a fast, comprehensive, and intuitive manner but still requires a costly operating system. VIVIAN system substantially contributed to surgical planning by 1) providing a quick and better understanding of intracranial anatomic and abnormal spatial relationships, 2) simulating the craniotomy and the required cranial base bone work, and 3) simulating intraoperative views.
Hohlweg-Majert, B. [31]	2005	Dual (Baseline, pre-surgery and post-surgery as clinically indicated	Spiral-CT Contrast-enhanced MRI	n/a	Author’s opinion without data analysis: Image-guided treatment improves preoperative planning by visualizing the individual anatomy, and outlining the intended reconstructive outcome. Intraoperative navigation makes tumor and reconstructive surgery more reliable by showing safety margins, saving vital structures, and leading the reconstruction to preoperatively planned objectives.
Qiu TM [32]	2010	Dual (Baseline and after 6 months)	3.0-T whole-body MRI (General Electric Medical Systems, GE Signa VH/i) Diffusion tensor imaging (DTI), T1-weighted 3-D fast spoiled gradient-recalled	n/a	VR-based 3D stereoscopic tractography visualization enhances understanding of anatomic information of intra-axial tumor contours and adjacent PT and results in surgical trajectory optimization initially and maximal safe tumor resection finally. Surgeons can predict the long-term motor functional outcome based on EPT increasing amplitude.
Yang, JH [33]	2019	Single	MRI	n/a	Positive effects of VR experience of 3D reconstructed knee MRIs in patients undergoing arthroscopic knee surgery reducing anxiety around surgical encounters.

**Table 5:** Technical Setup and Intervention Type.

First Author	Year of Publication	Measurements	Hardware	Software	Pre-Surgical or Intervention
Pediatric only (N = 7)					
Ieiri S [19]	2012	Mean +/− SD of fiducial registration error (FRE) and target registration error (TRE) between the lists of markers identified in the CT images for evaluation of registration accuracy	Mean +/− SD of fiducial registration error (FRE) and target registration error (TRE) between the lists of markers identified in the CT images for evaluation of registration accuracy	3D viewer software (Virtual Place 300, AZE Co., Ltd., Tokyo, Japan	Intervention
Souzaki R [20]	2013	Descriptive report of success or not of tumor resection using the AR navigation	Descriptive report of success or not of tumor resection using the AR navigation	3D viewer software program (Virtual Place 300, AZE Co., Ltd.	Pre-surgical
Zhao C [21]	2015	Descriptive characteristics and degree of obstruction of parts of the ventricular system (complete vs. non-complete obstruction) by MRVE and ventriculoscopy	Descriptive characteristics and degree of obstruction of parts of the ventricular system (complete vs. non-complete obstruction) by MRVE and ventriculoscopy	Processing software (ADW 4.3) for Signa EXCTTE Smooth imaging Navigator software	Intervention
Han, S-H. [22]	2019	Anxiety and distress: Observational Scale of Behavioral Distress (OSBD) (0–30 scale) Need for parental presence (yes/no) Parental satisfaction score (0–10 scale) Procedure time (seconds from room entry to image capture). Number of repeated radiographs Process difficulty score	Oculus Go VR headset	Custom VR content developed by JSC Games (Seoul, Republic of Korea) Randomization software: Random Allocation Software (version 1.0, Isfahan University of Medical Sciences). Statistical analysis software: SPSS (version 21.0, SPSS Inc).	Pre-intervention: VR was used before chest radiography to educate and prepare pediatric patients
Milano, E.G. [23]	2021	3D visualization, LVOT max, RVOT max	Models were printed at 1:1 scale, in rigid white nylon (EOS PA2200 Nylon 12) using selective laser technology (EOS P100).	The 3D reconstructions (as .obj files) were imported into a novel VR environment developed in-house within the Unity engine. The target platform was the Oculus Rift system (comprising a headset, two sensors, and two hand controllers).	Pre-surgical
Stunden, C. [24]	2021	MoTrak head motion tracking system, Venham picture test, short State-Trait Anxiety Inventory, procedural data (minutes), usability, child reported satisfaction and fun	MERGE VR Headset, Samsung Galaxy S9	Unity, AirServer Connect	Pre-surgical
Ryu, J-H [25]	2021	Anxiety and distress: Observational Scale of Behavioral Distress (OSBD) (0–30 scale) Need for parental presence (yes/no) Parental satisfaction score (0–10 scale) Number of repeated radiographs Process difficulty score Ease-of-procedure score (0–10 scale rated by radiology technologists)	VR group: Head-mounted VR display Tablet group: Tablet PC for video education	Custom VR content developed based on a simulated radiology room experience Tablet video was 2D conversion of the same VR material Randomization software: Random allocation software (version 1.0, Isafahan University of Medical Sciences) Statistical analysis software: SPSS (version 21.0, SPSS Inc.)	Pre-intervention: VR was used before chest radiography to educate and prepare pediatric patients
Adult only (N = 4)					
Simpfendorfer T [26]	2016	Descriptive feasibility and performance assessed by histopathological results, peri- and postoperative data.	Descriptive feasibility and performance assessed by histopathological results, peri- and postoperative data.	Open-source Medical Imaging Interaction Toolkit (MITK) syngo iPilot; Siemens Health-care,	Intervention
Nambi G [27]	2020	Imaging (muscle cross-sectional area, muscle thickness) Biochemical (CRP, TNF,-alpha, IL-2, IL-4, IL-6)	Imaging (muscle cross-sectional area, muscle thickness) Biochemical (CRP, TNF,-alpha, IL-2, IL-4, IL-6)	VR: ProKin system (PK 252, N Tecno Body, Italy)	Intervention
Sadri, S [28]	2023	Primary outcomes measured include iodinated contrast volume, times to filter placement, and fluoroscopy time. Secondary outcomes involve creatinine levels at discharge and clinical adverse events such as stroke and death at 30-day follow-up.	Microsoft HoloLens	Unity, 3mensio	Intervention
Chuan, A [29]	2023	Needling performance scores, calculated based on needle angulation (ideal in-plane positioning), number of withdrawals (fewer is better), time taken to complete the task Presence Questionnaire (for immersion in the virtual environment) NASA Task Load Index (NASA-TLX) (for cognitive workload)	VR Headset: Oculus Rift S Hand controllers (to manipulate virtual transducer and needle) Motion Capture System: MTw Awinda (Xsens, The Netherlands) Ultrasound Machine: Edge (FujiFilm SonoSite, Bothell, WA, USA) Computer: Gaming laptop with Intel Core i7-8750H processor (2.2 GHz) 16GB RAM NVIDIA GeForce GTX 1060 graphics card	Unity C (for VR trainer development) SPSS version 24 (for statistical analysis) MedCalc version 20.115 Microsoft Excel (2009 version) NASA-TLX software (iOOS version 1.03) for cognitive workload measurement	Pre-surgical (VR trainer was designed for training in ultrasound guided regional anesthesia (UGRA), not for direct intervention on patients
Pediatric and adult (N = 4)					
Kockro R, A. [30]	2000	Descriptive analysis: A report was written about each planning session. Each report was divided into two parts. One part recorded the size of the data set, the time needed for fusion and segmentation, the average system performance in frames per second, and the planning and simulation tools used during the session.	Descriptive analysis: A report was written about each planning session. Each report was divided into two parts. One part recorded the size of the data set, the time needed for fusion and segmentation, the average system performance in frames per second, and the planning and simulation tools used during the session.	VIVIAN (Virtual Intracranial Visualization and Navigation)	Pre-surgical
Hohlweg-Majert, B. [31]	2005	Pre- and postoperative spiral CT/MRI changes between the preceding and actual CT/MRI (e.g., assessment of pre- and post-operative periorbital deformation, optic nerve decompression).	Pre- and postoperative spiral CT/MRI changes between the preceding and actual CT/MRI (e.g., assessment of pre- and post-operative periorbital deformation, optic nerve decompression).	VR surgical navigation tool (Stryker-Leibinger, Germany)	Pre-surgical
Qiu TM [32]	2010	Karnofsky Performance Scale (KPS): grading functional status at 6-month evaluation, patient questionnaire (at follow-up)	Karnofsky Performance Scale (KPS): grading functional status at 6-month evaluation, patient questionnaire (at follow-up)	VR system (Dextroscope, Volume Interactions Pte. Ltd.)—the Fiber Tracking module of the VR system Volume-One 1.72 (VOLUME-ONE developers group) dTV. II (Image Process and Analysis Laboratory	Pre-surgical
Yang, JH [33]	2019	Primary: Amsterdam Preoperative Anxiety and Information Scale score to measure level of anxiety; Secondary: visual analog scale (VAS) scores measuring patient pain, preparedness, satisfaction, and stress	Primary: Amsterdam Preoperative Anxiety and Information Scale score to measure level of anxiety; Secondary: visual analog scale (VAS) scores measuring patient pain, preparedness, satisfaction, and stress	Software 3D Slicer 4.6.2 (Brigham and Women’s Hospital and The Slicer Community Autodesk 3ds Max (Autodesk ZBrush (Pixologic) Engine 4 Software (Epic Games)	Intervention

**Table 6:** Standard for Reporting of Diagnostic Accuracy (STARD) consensus scores for primary studies of this review.

Study	Items Reported (Out of 19)	Final Consensus Score (Maximum = 19 *)	% Score **	Quality
Pediatric only (N = 7)				
Ieiri 2012 [19]	19	10	53%	Fair
Souzaki 2013 [20]	19	9.5	50%	Suboptimal
Zhao 2015 [21]	19	12.5	66%	Good
Han 2019 [22]	19	16	84%	Excellent
Milano 2021 [23]	19	13	68%	Good
Stunden 2021 [24]	18	16	89%	Excellent
Ryu 2021 [25]	19	15	79%	Good
Adult only (N = 4)				
Simpfendorfer 2016 [26]	19	10	53%	Fair
Nambi 2020 [27]	19	16.5	87%	Excellent
Sadri 2023 [28]	19	13.5	71%	Good
Chuan 2023 [29]	19	14	74%	Good
Pediatric and adult (N = 4)				
Kockro 2000 [30]	19	11.5	61%	Fair
Hohlweg-Majert 2005 [31]	19	8	42%	Suboptimal
Qui 2010 [32]	19	15	79%	Good
Yang 2019 [33]	19	17	89%	Excellent

* Maximum = score of 19, except for Stunden [24], maximum score = 18. ** % score [consensus score × 100]/maximum score (=19, except for Stunden [24] = 18)].

## Data Availability

The authors used data from the selected primary studies as available in the public domain. For information about raw data of individual primary studies of this review, we encourage the readers to contact the authors of the primary studies directly.

## Supplementary Materials

The following supporting information can be downloaded at: https://www.mdpi.com/article/10.3390/jcm14207438/s1, Table S1: Standard for Reporting of Diagnostic Accuracy (STARD) consensus scores for primary studies of this review with breakdown consensus scores of items per study.
