# Supplementary material for: Clinical Applications of Virtual and Augmented Reality in Radiology: A Scoping Review
Source: J Clin Med. 2025 Oct 21;14(20):7438. doi: 10.3390/jcm14207438 (PMC12565096; doi:10.3390/jcm14207438)
Supplement: Supplementary file 1 [file jcm-14-07438-s001.zip › jcm-3810616-supplementary.pdf]

Supplementary Table S1. Standard for Reporting of Diagnostic Accuracy (STARD) consensus scores for primary studies of this review with breakdown consensus scores of items per study.

STARD	Item 1	Item 2	Item 3	Item 4	Item 5
	Identification as a study of diagnostic accuracy using at least one measure of accuracy	Structured summary of study design, methods, results, and conclusions	Scientific and clinical background, including the intended use and clinical role of the index test	Study objectives and hypotheses	Whether data collection was planned before the index test (and reference standard) was performed (prospective study) or after (retrospective study)
Score 1	Provides information about ROC, predictive value, TP/ FP; TN, FN; Sensitivity, Specificity, correlation, reliability, responsiveness				
Score 0.5		Non-structured abstract		1 of objectives or hypotheses	
Score 0	No key word	No abstract			
N/A					
Kockro, R [19]	0	1	1	1	1
Hohlweg-Majert B [20]	0	1	1	1	1

Qiu TM [21]	0	1	1	1	1
Ieiri S [22]	0	1	1	1	1
Souzaki R [23]	0	1	1	1	1
Zhao C [24]	0	1	1	1	1
Simpfendorfer T [25]	0	1	1	1	1
Yang JH [26]	0	1	1	1	1
Han S-H [27]	0	1	0	1	1
Nambi G [28]	0	1	1	1	1
Milano E [29]	0	1	1	1	1
Stunden C [30]	0	1	1	1	1
Ryu J-H [31]	0	1	0	1	1
Sadri S [32]	0	1	1	1	1
Chuan A [33]	0	1	0	1	1

	Item 6	Item 7	Item 8	Item 9	Item 10a
	Eligibility criteria	On what basis potentially eligible participants were identified (such as symptoms, results from previous tests, inclusion in registry)	Where and when potentially eligible participants were identified (setting, location and dates)	Whether participants formed a consecutive, random or convenience series	Index test, in sufficient detail to allow replication
Score 1	Must have at least one inclusion/exclusion criterion, and location of study.	Reports the test/examination used to identify the subjects (needs to be for study purposes)			Provides details that enable the reader to reproduce all tests, except for information about operators

Score 0.5	One of inclusion/exclusion criteria, orlocation of study	Non-structured abstract			
Score 0	Missing inclusion/exclusion criteria and location.	No abstract		Not reported	When there is not enough details for readers to reproduce the scans
N/A					
Kockro, R [19]	1	1	0.5	0	1
Hohlweg-Majert B [20]	1	1	1	0	0.5
Qiu TM [21]	1	1	1	1	1
Ieiri S [22]	0	1	0	0	1
Souzaki R [23]	0	0	0	0	1
Zhao C [24]	1	1	1	1	1
Simpfendorfer T [25]	0	1	0	0	1
Yang JH [26]	1	1	1	1	1
Han S-H [27]	1	1	1	1	1
Nambi G [28]	1	1	0.5	1	1
Milano E [29]	1	1	1	0	1
Stunden C [30]	1	1	1	1	1
Ryu J-H [31]	1	1	1	1	1
Sadri S [32]	1	1	1	0	1
Chuan A [33]	1	1	1	1	1

	Item 18	Item 19	Item 20	Item 21a	Item 26
	Intended sample size and how it was	Flow of participants, using a diagram	Baseline demographic and clinical characteristic	Distribution of severity of disease in	Study limitations, including sources of

	determined		s of participants	those with the target condition	potential bias, statistical uncertainty, and generalisability
Score 1			Only 2 of the above	Spectrum of (primary) disease severity or subtypes of the disease (if the study recruits based on the primary disease then needs to report severity of primary disease)	
Score 0.5		Flow chart,but no numbers	Only 1 of the above		
Score 0			None	Not enough details to score 1	
N/A	When the research question does not include criterion validity (eg. comparison study where they don't indicate				

	the reference standard)				
Kockro, R [19]	0	0	1	1	0
Hohlweg-Majert B [20]	0	0	0.5	0	0
Qiu TM [21]	0	0	1	1	1
Ieiri S [22]	0	0	1	0	0
Souzaki R [23]	0	0	1	0.5	1
Zhao C [24]	0	0	0.5	1	1
Simpfendorfer T [25]	0	0	1	1	0
Yang JH [26]	0	1	1	1	1
Han S-H [27]	1	1	1	0	1
Nambi G [28]	1	1	1	1	1
Milano E [29]	0	0	1	1	1
Stunden C [30]	1	1	1	N/A (healthy volunteers)	1
Ryu J-H [31]	1	1	1	0	1
Sadri S [32]	0	0	1	0.5	1
Chuan A [33]	1	0	1	0	1

	Item 27	Item 28	Item 29	Item 30	Total /19
	Implications for practice, including the intended use and clinical role of the index test	Registration number and name of registry	Where the full study protocol can be accessed	Sources of funding and other support; role of funders	
Score 1					
Score 0.5					
Score 0					
N/A					
Kockro, R [19]	1	0	0	1	11.5/19
Hohlweg-Majert B [20]	0	0	0	0	8/19
Qiu TM [21]	1	1	0	1	15/19

Ieiri S [22]	1	0	1	1	10/19
Souzaki R [23]	1	1	0	0	9.5/19
Zhao C [24]	1	0	0	0	12.5/19
Simpfendorfer T [25]	1	0	0	1	10/19
Yang JH [26]	1	1	1	1	17/19
Han S-H [27]	1	1	1	1	16/19
Nambi G [28]	1	1	0	1	16.5/19
Milano E [29]	1	0	0	1	13/19
Stunden C [30]	1	1	1	0	16/18
Ryu J-H [31]	1	1	0	1	15/19
Sadri S [32]	1	1	0	1	13.5/19
Chuan A [33]	1	1	0	1	14/19

Note: STARD items excluded (N=15) due to the need of a reference standard / diagnostic accuracy measure which were not available on the selected primary studies of this scoping review:

Methods - Test Methods

10b: Reference standard, in sufficient detail to allow replication.

11: Rationale for choosing the reference standard (if alternatives exist).

12a: Definition of and rationale for test positivity cut-offs or result categories of the index test, distinguishing pre-specified from exploratory.

12b: Definition of and rationale for test positivity cut-offs or result categories of the reference standard, distinguishing pre-specified from exploratory.

13a: Whether clinical information and reference standard results were available to the performers/readers of the index test.

13b: Whether clinical information and index test results were available to the assessors of the reference standard.

Methods - Analysis

14: Methods for estimating or comparing measures of diagnostic accuracy.

15: How indeterminate index test or reference standard results were handled.

16: How missing data on the index test and reference standard were handled.

17: Any analyses of variability in diagnostic accuracy, distinguishing pre-specified from exploratory.

Results - Participants

21b: Distribution of alternative diagnoses in those without the target condition.

22: Time interval and any clinical interventions between index test and reference standard.

Results – Test Results

23: Cross tabulation of the index test results (or their distribution) by the results of the reference standard.

24: Estimates of diagnostic accuracy and their precision (such as 95% confidence intervals).

25: Any adverse events from performing the index test or the reference standard.
